# Protein-Mediated and RNA-Based Origins of Replication of Extrachromosomal Mycobacterial Prophages

**DOI:** 10.1128/mBio.00385-20

**Published:** 2020-03-24

**Authors:** Katherine S. Wetzel, Haley G. Aull, Kira M. Zack, Rebecca A. Garlena, Graham F. Hatfull

**Affiliations:** a Department of Biological Sciences, University of Pittsburgh, Pittsburgh, Pennsylvania, USA; Weill Cornell Medical College

**Keywords:** *Mycobacterium*, bacteriophage genetics, bacteriophages

## Abstract

Temperate bacteriophages are common and establish lysogens of their bacterial hosts in which the prophage is stably inherited. It is typical for such prophages to be integrated into the bacterial chromosome, but extrachromosomally replicating prophages have been described also, with the best characterized being the Escherichia coli phage P1 system. Among the large collection of sequenced mycobacteriophages, more than half are temperate or predicted to be temperate, most of which code for a tyrosine or serine integrase that promotes site-specific prophage integration. However, within the large group of 621 cluster A temperate phages, ∼20% lack an integration cassette, which is replaced with a *parABS* partitioning system. A subset of these phages carry genes coding for a RepA-like protein (RepA phages), which we show here is necessary and sufficient for autonomous extrachromosomal replication. The non-RepA phages appear to replicate using an RNA-based system, as a *parABS*-proximal region expressing a noncoding RNA is required for replication. Both RepA and non-RepA phage-based plasmids replicate at one or two copies per cell, transform both Mycobacterium smegmatis and Mycobacterium tuberculosis, and are compatible with pAL5000-derived *oriM* and integration-proficient plasmid vectors. Characterization of these phage-based plasmids offers insights into the variability of lysogenic maintenance systems and provides a large suite of plasmids for actinobacterial genetics that vary in stability, copy number, compatibility, and host range.

## Abstract (precis)

Bacteriophages are the most abundant biological entities in the biosphere and are a source of uncharacterized biological mechanisms and genetic tools. Here, we identify segments of phage genomes that are used for stable extrachromosomal replication in the prophage state. Autonomous replication of some of these phages requires a RepA-like protein, although most lack *repA* and use RNA-based systems for replication initiation. We describe a suite of plasmids based on these prophage replication functions that vary in copy number, stability, host range, and compatibility. These plasmids expand the toolbox available for genetic manipulation of *Mycobacterium* and other *Actinobacteria*, including Gordonia terrae.

## INTRODUCTION

Bacteriophages are the most abundant biological entities in the biosphere and are a source of vast genetic diversity (1). Mainly through the Science Education Alliance Phage Hunters Advancing Genomics and Evolutionary Science (SEA-PHAGES) program, more than 17,000 bacteriophages infecting hosts of the phylum *Actinobacteria* have been isolated, of which more than 3,000 have been sequenced (https://phagesdb.org). These bacteriophages can be sorted into related groups (clusters A, B, C, etc.) according to their overall relatedness (2, 3), and ∼50% of these contain phages that are likely to be temperate, coding for predicted repressor and integrase genes (4). Two classes of integrases have been described—tyrosine and serine integrases—that are used to integrate the phage genome into the host chromosome when establishing lysogeny. This enables the prophage to be passively replicated with the host genome and ensures that a prophage is present in each of the daughter cells after division. Although integration systems are well studied and common among temperate phages (5–7), some temperate phages, including the prototype Escherichia coli phage P1, maintain their prophages extrachromosomally, and carry genes coding for components of partitioning and recombination systems that ensure prophage maintenance (8). “Plasmidial” prophages (9) are relatively uncommon but have been reported for diverse bacteria, including Bacillus anthracis (10), Borrelia burgdorferi (11), Chlamydia pneumoniae (12), and Staphylococcus aureus (9), in addition to P1-family phages (13) and the linearly replicating phage N15 (14). Extrachromosomal prophages are likely underrepresented in genome sequencing projects (15).

Partitioning systems have been reported for cluster A temperate mycobacteriophages, including CRB1 and RedRock (16, 17), that presumably facilitate prophage maintenance (18). RedRock lacks an integrase gene, but carries genes coding for a *parABS* system and replicates extrachromosomally with a prophage average copy number of 2.4 copies/cell (16). RedRock ParB is a DNA-binding protein and recognizes two *parS* loci (*parS-L* and *parS-R*), each of which contains eight directly repeated copies of an 8-bp motif. RedRock *parA* and *parB* are expressed lysogenically as expected, and the *parABS* cassette stabilizes extrachromosomally replicating shuttle plasmids such as those based on *oriM* from Mycobacterium fortuitum plasmid pAL5000 (16).

Extrachromosomal maintenance requires a system for initiation of DNA replication. In phage P1, an initiator protein, RepA and an origin of replication is required, as well as ParA and ParB (reviewed in reference 19); related systems have been described for prophages of ΦHAP-1 of Halomonas aquamarina (20), pVv01 of Vibrio vulnificus (21) and lcp3 of Leptospira interrogans (22). Replication initiator protein genes are commonly found in plasmids, and putative replication initiator protein open reading frames (ORFs) have been identified in several naturally occurring mycobacterial plasmids, including plasmids pLR7 (Mycobacterium avium) (23), pJAZ38 (Mycobacterium fortuitum) (24), pCLP (Mycobacterium celatum) (25), and pMF1 (M. fortuitum) (26). The replication cassette commonly used in plasmids for genetic manipulation of mycobacteria (*oriM*) derives from M. fortuitum plasmid pAL5000, which has a copy number in Mycobacterium smegmatis of about 23 (27). The replication cassette genes encode two proteins that are required for replication, RepA and the DNA-binding protein, RepB (28). Mycobacteriophage DNA replication systems are not well-characterized for either lytic growth or extrachromosomal replication.

Many different plasmid replication systems have been described (29), and although most require an initiator protein, some require only RNA products for initiation and copy number regulation. The most common example is the replication cassette of E. coli plasmid ColE1, the basis for many plasmids used in recombinant DNA, including pBR322, pUC18/19, and their derivatives (30). In these systems, an RNA molecule (RNA II) acts as a primer for DNA replication by the host DNA polymerase I (Pol I), and a second RNA (RNA I) modulates its activity to determine copy number (31). We previously suggested that the *parABS* mycobacteriophage RedRock might use an RNA-based replication system for prophage replication, as a noncoding RNA is expressed adjacent to the *parABS* cassette, and no *repA* homologue or similar gene in the genome was identified (16). However, we were not able to demonstrate autonomous replication by a DNA cassette containing this region. In contrast, mycobacteriophage CRB1 codes for a putative RepA protein (17).

Here, we characterize the prophage origins of replication for eight temperate mycobacteriophages: Miko, Rachaly, Jeeves, RedRock, Alma, Gladiator, Et2Brutus, and LadyBird. Miko, Rachaly, and Jeeves prophages initiate replication with a RepA-like replication initiator protein, but RedRock, Alma, Gladiator, Et2Brutus, and LadyBird use an initiator RNA, and to our knowledge, these are first RNA-based prophage replication systems. Plasmids that include these origins vary in copy number, retention without selection, and compatibility in M. smegmatis mc^2^155, and differ in functionality in Mycobacterium tuberculosis and Gordonia terrae. Plasmids based on these prophage origins broaden the suite of tools available for genetic manipulation of *Actinobacteria*.

## RESULTS

### Spectrum of extrachromosomally replicating actinobacteriophage prophages.

**FIG 1:**
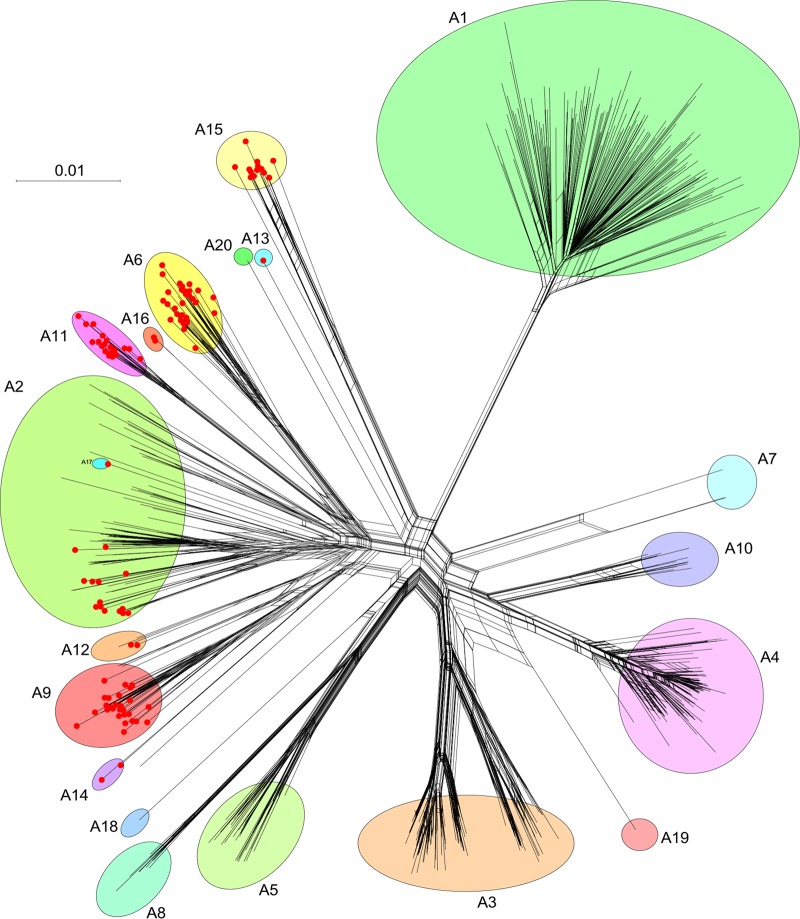
Network phylogeny of cluster A mycobacteriophages. A network phylogeny of 621 cluster A mycobacteriophages was constructed based on gene content and represented using Splitstree (58). A database “Actino_Draft” dated 9 December 2019 was used in which predicted gene products were sorted into groups (phamilies) of related sequences as described previously (1, 59) (C. Gauthier and G. F. Hatfull, unpublished data), and a nexus-formatted file generated using the custom script “PhamNexus.” Colored circles illustrate the 20 subclusters (A1 to A20), and red dots at nodes indicate phages carrying *parABS* systems. The bar indicates the number of substitutions per site.

**FIG 2:**
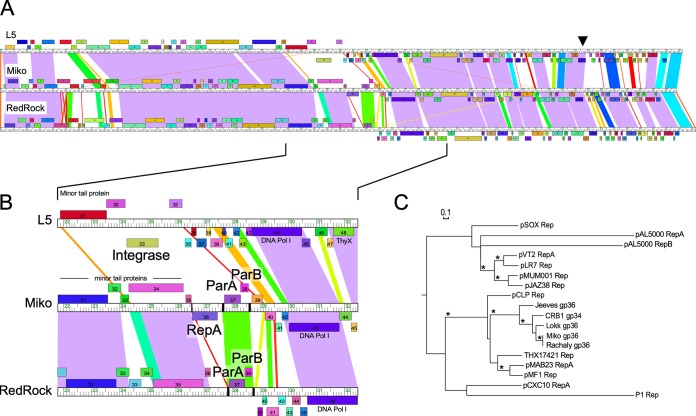
Alignment of *parABS* phages used in this study. (A) Representative RepA (Miko) and non-RepA (RedRock) phage genomes were aligned to that of the integrating phage L5. The ruler shows genome length in kilobases, while ORFs shown above and below the ruler are transcribed rightward and leftward, respectively. ORFs of the same color have been assigned to the same gene phamily. A black arrowhead indicates the location of the immunity repressor gene in all three genomes. Shared nucleotide sequence similarity is represented as spectrum-colored shading, with violet representing the most similar and red the least similar above a BLASTN E-value threshold of 10^−4^. (B) Genome segments containing genes relevant for prophage replication and maintenance. Where present, integrase (L5), homologues to *repA* (Miko), *parA* and *parB* (Miko and RedRock) are labeled. The locations of centromere-like *parS* sites are indicated in the Miko and RedRock genome rulers with black bars. DNA Pol I, DNA polymerase I. (C) Relatedness of the phage RepA proteins to replication initiator proteins found in actinobacterial plasmids and the E. coli phage P1, shown as a phylogeny generated by maximum likelihood (PhyML). Bootstrap values greater than 70% are indicated with an asterisk, and the bar indicates the number of substitutions per site.

The number of sequenced actinobacteriophages has increased substantially over the past 5 years, including a 3.5-fold increase in the number of cluster A phages (32). Reexamination of the sequenced genomes reveals an expanded set of 110 “*parABS*” phages (see Table S1 in the supplemental material) grouped in the large 621-member cluster A. However, the cluster A phages are a highly diverse group, and they can be divided into 20 subclusters (Fig. 1). The largest subcluster, A1, is devoid of *parABS* phages, and all phages contain either a tyrosine or serine integrase (33); there are also no *parABS* phages in subclusters A3, A4, A5, A7, A8, A10, A18, A19, or A20 (Fig. 1). In contrast, all of the members of subclusters A6, A11, A13, A14, A15, A16, and A17 have a *parABS* system, together with 27 of the 31 subcluster A9 phages, two of the four subcluster A12 phages, and 15 of the 90 subcluster A2 phages (Fig. 1). Of the 110 *parABS* phages, 97 infect Mycobacterium smegmatis mc^2^155, and 13 infect Gordonia terrae 3612 (https://phagesdb.org), although all of the *Gordonia parABS* phages are in subcluster A15 (Fig. 1). In all 110 phages, the *parABS* cassette is centrally located in the viral genome, in a colinear position to the integration cassettes of closely related genomes (Fig. 2A). All of the *parA* proteins appear to be homologues and are grouped into a single protein “phamily” (3). However, there is considerable diversity among the *parB* proteins, which fall into at least four distinct protein phamilies. Three of these are represented in the 15 subcluster A2 *parAB* phages, and it is likely that *parB* is under selection to diversify to avoid incompatibility.

10.1128/mBio.00385-20.1 TABLE S1 Cluster A actinobacteriophages that contain *parABS.* Download Table S1, DOCX file, 0.02 MB. Copyright © 2020 Wetzel et al. 2020 Wetzel et al. This content is distributed under the terms of the Creative Commons Attribution 4.0 International license.

### Identification of putative prophage origins of replication.

In the *parABS* phages, substitution of the integrase functionality requires not only *parABS* partitioning functions but also the functions needed to promote the initiation of extrachromosomal DNA replication and copy number control. Genome alignments suggest that the replication and partitioning functions of the *parABS* phages must be closely linked and centrally located, downstream of the virion structure and assembly genes (Fig. 2). Using genome alignments, we identified two subsets of *parABS* phages. First, the “RepA” phages which carry genes encoding homologues of RepA plasmid initiation proteins located to the left of the *parABS* cassette, and second, the “non-RepA” phages for which RepA homologues have not been identified (Table S1). The non-RepA phages, including RedRock, represent the vast majority of *parABS* phages (95%; 105/110) and have representatives in each of the subclusters containing *parABS* phages. Five phages code for a RepA-like protein, four in subcluster A2 (Miko, Rachaly, Lokk, and CRB1), and one (Jeeves) of the two subcluster A14 *parABS* phages (Fig. 1; Table S1). In each of these, *repA* is transcribed in the opposite direction to both *parABS* and the virion structure and assembly genes (Fig. 2).

The five phage-encoded RepA-like proteins are 320 to 350 residues long and share ∼40% conserved amino acid residues. Miko and Rachaly RepA are very closely related (98% amino acid identity), but distantly related to Jeeves RepA, to which they share only ∼50% amino acid identity. Database searches strongly support the functional assignment of these phage-encoded RepA proteins. For example, Jeeves RepA is related to a plasmid-encoded RepA in Mycobacterium abscessus plasmid pMAB23 (34) (46% identity over 250 residues), as well as RepA proteins found in mycobacterial plasmids of the pMSC262 family, such as plasmid pCLP (Mycobacterium celatum) and plasmid pMF1 (M. fortuitum) (25, 26). Related proteins are also present in genome assemblies of several *Mycobacterium* species, including M. abscessus, Mycobacterium cosmeticum, and Mycobacterium tusciae (e.g., NCBI:protein accession number TXH17421; Fig. 2C). The mycobacteriophage RepA proteins are not closely related to RepA proteins of pMSC262 family members pLR7 and pJAZ38 (23, 24, 35) or to RepA and RepB encoded by pAL5000 (36). The phage RepA proteins contain an N-terminal predicted helix-turn-helix DNA-binding domain (Miko residues 95 to 132), a common feature of plasmid replication initiator proteins (31).

Well-characterized plasmid replication proteins typically bind to either direct repeat sequences (“iterons,” as in RepA of phage P1 [37]), palindromic sequences (e.g., RepB of pAL5000 *oriM*), or conserved sequence motifs (e.g., plasmids pMF1, pCLP, pJAZ38, pLR7, and pMSC262) at the origin of replication; these are typically tightly linked to the *repA* gene (26, 31). We have not identified repeated sequence motifs in the regions between the virion tail genes and *parS-L* but note that nucleotide sequence conservation extends ∼50 bp upstream of the *repA* gene in Rachaly, Miko, Lokk, and CRB1. It thus seems likely that the origin of replication lies either immediately upstream of *repA* or within the *repA* gene itself, similar to the position of the phage lambda origin within the *o* gene (38, 39). We note that the RepA phages Miko and Rachaly differ from the previously characterized non-RepA *parABS* phages (RedRock, Alma, Et2Brutus, Gladiator, and LadyBird [16]) in having an additional *parS* locus containing five direct repeats, immediately downstream of the *repA* gene (see Fig. 5). In phages Lokk and CRB1, there is no intergenic space between the 3′ ends of *repA* and the adjacent rightward-transcribed gene and no additional *parS* site. We note that Jeeves, Lokk, and CRB1 appear to lack *parS-R* (see Fig. 5), which had been observed for several other *parABS* phages (16).

### Copy numbers of non-RepA prophages.

We previously determined that the prophage copy number in a lysogen of the non-RepA phage RedRock was 2.4 copies/cell (16); here we extended this analysis to include additional non-RepA lysogens of Alma, Et2Brutus, and LadyBird. DNA was extracted from lysogens and sequenced, and the ratio of sequence reads mapping to the bacterial chromosome and the prophage genome was calculated (Table 1). Because the sample also contains phage DNA from spontaneous lytic induction, the proportion of prophage-derived reads was derived from the ratio of sequence reads traversing the cohesive ends (i.e., from prophages) relative to those corresponding to genome cleaved at cos during packaging (i.e., from packaged genomes). The adjusted prophage copy numbers were 4.8, 3.7, and 2.5 for Alma, Et2Brutus, and LadyBird, respectively (Table 1). These copy numbers may be slightly overestimated, because some reads across genome ends could be derived from unpackaged concatemers during lytic replication. However, ratios of ligated to cleaved *cos* sites as high as 3:1 (Et2Brutus; Table 1) are unlikely to solely indicate lytic growth and are more consistent with extrachromosomally replicating prophages. We were not able to measure copy numbers for prophages of Miko, Rachaly, and Jeeves due to somewhat higher levels of spontaneous lytic induction.

**TABLE 1:** Copy numbers of extrachromosomal prophages

Strain(phage)	Phage reads^*a*^	Precise end reads^*b*^	End- spanning reads^*c*^	Total end reads	End- spanning reads: total end reads	Coverage		Phage/host coverage	
						Phage	mc^2^155	Raw	Corrected^*d*^
mc^2^155(Alma)	59,386	41	46	87	0.53	167.5	18.5	9.1	4.80
mc^2^155(Et2Brutus)	32,465	11	35	46	0.76	92.8	19.2	4.8	3.69
mc^2^155(LadyBird)	27,265	11	19	30	0.63	77.0	19.1	4.0	2.55

a Sequence reads mapping to the phage genome out of a total of ∼1 million per sample.

b Sequence reads beginning at precisely the terminus of viral genomic DNA.

c Sequence reads that span the predicted 5′ and 3′ ends of the genomes.

d The ratio of phage/host genome coverage multiplied by the end-spanning reads:total end reads.

### Miko *repA* is required for prophage replication.

**FIG 3:**
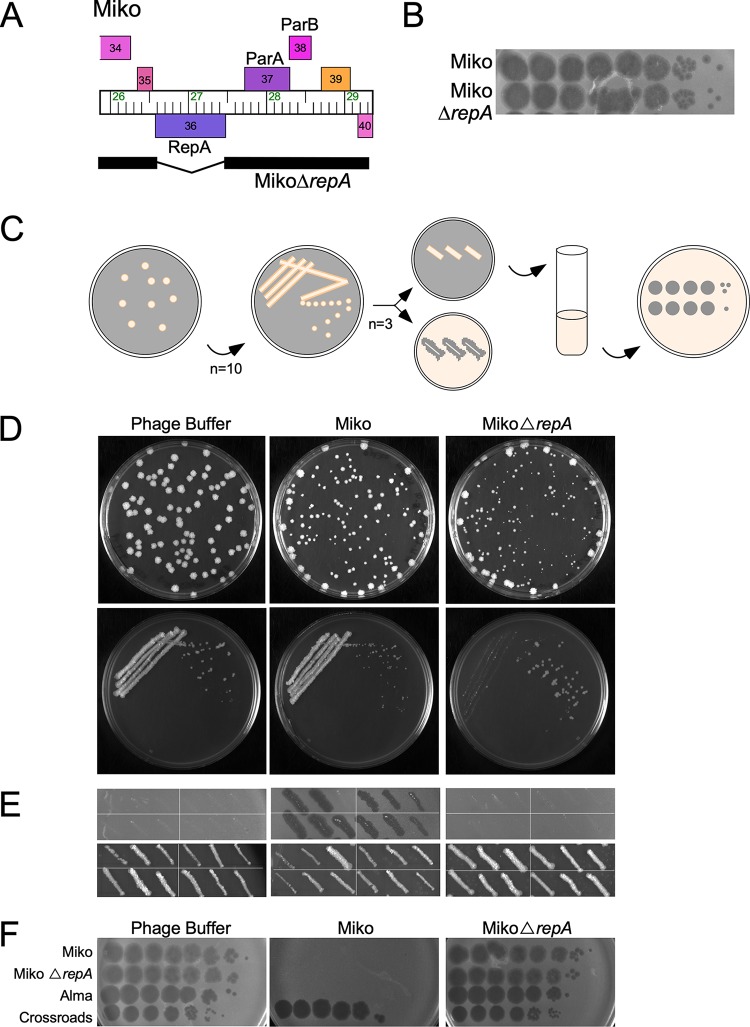
RepA is necessary to form stable lysogens of Miko. (A) Genome of MikoΔ*repA* mutant phage. Miko was engineered to remove *repA*, and the black bar shows the retained region (deletion coordinates 26597 to 27466). (B) Titer and plaque morphology of MikoΔ*repA* mutant phage. Lysates of Miko and Miko*ΔrepA* were 10-fold serially diluted and plated onto a lawn of M. smegmatis mc^2^155. (C) Scheme to characterize Miko*ΔrepA* lysogens. Lysogens were recovered by plating exponentially growing M. smegmatis mc^2^155 onto solid media seeded with phage buffer, Miko, or Miko*ΔrepA*. Ten individual colonies were streaked onto solid media to remove phage particles carried over from the selection plate. Three colonies from each streak plate were patched onto solid media and M. smegmatis lawns to test for spontaneous phage release. Liquid cultures were grown from these patches to test for phage superinfection. (D) Plates seeded with phage buffer, Miko, or Miko*ΔrepA* (top) and representative streaks from colonies grown on the plates (bottom). Larger colonies were recovered at the edges of the seeded plates where phage particles are likely less abundant were avoided. (E) Spontaneous phage release from four representative colonies grown in the presence of phage buffer, Miko, or Miko*ΔrepA.* None of the colonies recovered on media with phage buffer or MikoΔ*repA* released phages and are not stably lysogenic; at least two of the three purified streaks from colonies recovered on Miko-seeded plates are lysogenic and release phage particles. (F) Susceptibility of colonies to phage superinfection. Liquid cultures were grown from patches of 6 of the 10 colonies from the seeded plates and tested for their susceptibility to Miko, Miko*ΔrepA*, Alma, and a control phage Crossroads (L2). A representative example of each is shown.

To determine whether *repA* of phage Miko is required for prophage replication, we reasoned that *repA* deletion would have little or no effect on lytic growth but would lead to reduced lysogenic stability. A deletion derivative (MikoΔ*repA*) was constructed using an adaptation of bacteriophage recombineering of electroporated DNA (BRED) engineering (40) (Fig. 3A) and appears unaltered in its lytic properties; it can be readily propagated to high titer and has a plaque morphology similar to that of its parent phage (Fig. 3B). To test for lysogeny, a liquid culture of M. smegmatis mc^2^155 was diluted, and colonies were recovered on solid media seeded with Miko or MikoΔ*repA* (Fig. 3C). Similar numbers of colonies were recovered on Miko-seeded medium as with a buffer control, reflecting a high lysogenization frequency under these experimental conditions (Table 2). To confirm that the recovered derivatives are lysogenic for Miko, 10 individual colonies were restreaked to remove phage particles carried over from the selection plate and tested for phage release and superinfection immunity (Fig. 3D); nearly all (28/30) of the individual colonies picked from the restreaks spontaneously released phage (Fig. 3E; Table 2), and a tested subset were all immune to superinfection (Fig. 3F). Interestingly, a similar number of survivors was recovered on MikoΔ*repA*-seeded plates consistent with a high rate of lysogenization, although the colonies were smaller than those on the Miko-seeded plates, reflecting slowing growth (Table 2). When restreaked, the pattern of growth is distinctly different from those taken from the Miko-seeded plates (Fig. 3D); the densest part of the streak fails to grow well—presumably due to phage carryover and lytic phage replication—and the single colonies recovered are not lysogens when retested for phage release and immunity (Fig. 3E and F; Table 2). These data suggest that Miko *repA* is not required for establishment of lysogeny and immunity, but that it is required for stable lysogeny and prophage inheritance.

**TABLE 2:** Lysogens of MikoΔ*repA* and AlmaΔ*ori*

Phage or control	No. of colonies/plate	% frequency of colony formation^*a*^	Phage release from patch (3 per original colony)^*b*^	Colonies yielding ≥1 patch with phage release^*c*^
Miko	91	128	28	10
Miko△*repA*	100	140	0	0
Phage buffer	71	100	0	0
Alma	154	112	29	10
Alma△*ori*	158	114	11	7
Unseeded	138	100	0	0

a Relative to the colony recovery using only phage buffer.

b Ten colonies were picked from the seeded plates and restreaked, and three colonies of each were tested for phage release and immunity. The numbers of colonies of the 30 total colonies releasing phage are shown.

c The proportions of each of the original 10 colonies restreaked from seeded plates of which at least one of the three retested colonies released phage.

### Role of the putative replication origin of phages Alma and LadyBird in lysogeny.

**FIG 4:**
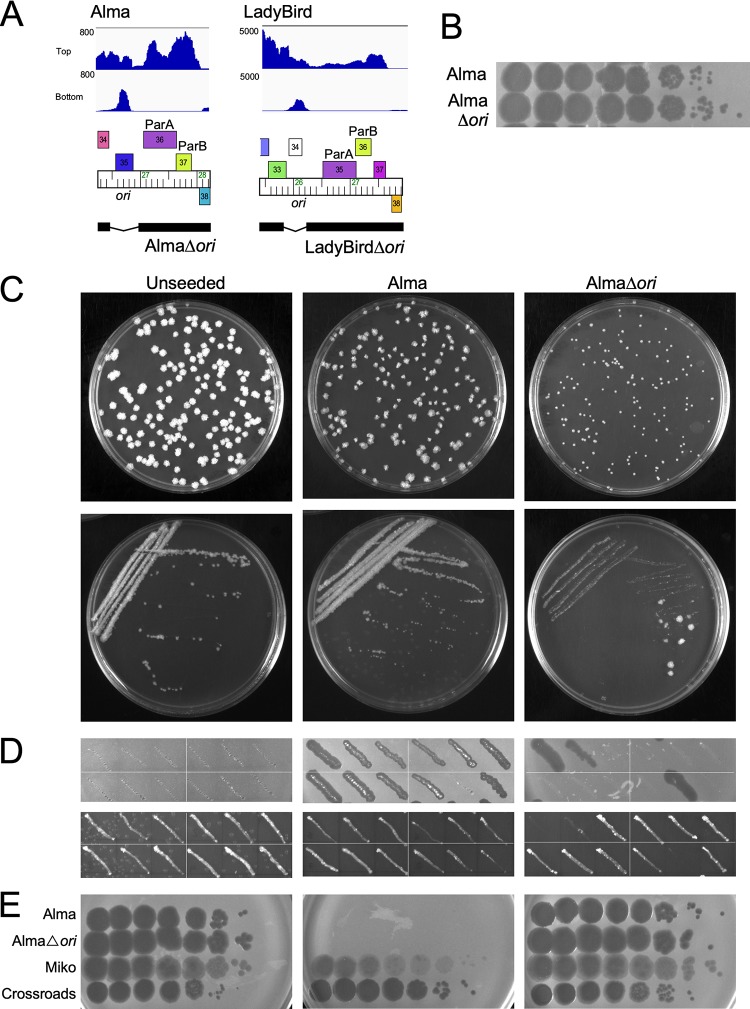
*ori* is necessary to form stable lysogens of Alma. (A) Genomes of Δ*ori* mutant phages. Phages were engineered to delete the noncoding RNA region (labeled *ori*) expressed by the prophage (Alma, LadyBird). The black bar below the genome map segment shows the retained regions. The coordinates of the deleted regions are 26463 to 26959 and 25900 to 26299 for Alma and LadyBird, respectively. Strand-specific RNA-Seq reads are aligned above the Alma and LadyBird maps. (B) Titer and plaque morphology of AlmaΔ*ori*. Lysates of Alma and AlmaΔ*ori* were 10-fold serially diluted and plated onto a lawn of M. smegmatis mc^2^155. (C) Characterization of Alma*Δori* lysogens (akin to Fig. 3C). Lysogens were recovered by plating serial dilutions of exponentially growing M. smegmatis mc^2^155 onto unseeded solid media or solid media seeded with Alma or AlmaΔ*ori* (Top). Ten (Alma, AlmaΔ*ori*) or six (unseeded) individual colonies were streaked onto solid media to remove phage particles carried over from the selection plate (bottom) (representative shown). (D) Spontaneous phage release from colonies grown in the presence of AlmaΔ*ori.* Three colonies from each streak plate were patched onto solid media and M. smegmatis lawns to test for spontaneous phage release. None of the colonies recovered on unseeded media released phage, but at least two of the three colonies from purified streaks from colonies recovered on Alma-seeded plates are lysogenic and release phage particles. Some patches originating from AlmaΔ*ori*-seeded plates released phage, while others did not. Patches that did not release phage grew well on solid media, while patches that did release phage grew poorly on solid media. (E) Susceptibility of colonies to phage superinfection. Liquid cultures were grown from patches of 6 of the 10 colonies from the seeded plates and tested for their susceptibilities to Alma, Alma*Δori*, Miko and a control phage Crossroads (L2). A representative example of each is shown.

Phages Alma and LadyBird lack a *repA* gene, but transcriptome sequencing (RNA-Seq) data for both show expression of RNA immediately upstream of *parA*, but in the reverse direction (Fig. 4A; see also Fig. S1 in the supplemental material). This RNA does not correspond to a predicted ORF, as we reported previously for several other non-RepA phages (16). We note that some of these phages have predicted ORFs in the forward direction that overlap with the noncoding RNA, a subset of which (e.g., Alma *35*) are also present in integrase-encoding cluster A phages and are unlikely to be involved in extrachromosomal replication (Fig. 4A). To explore whether these regions are required for prophage maintenance, we constructed deletion derivatives of Alma and LadyBird (AlmaΔ*ori* and LadyBirdΔ*ori*, respectively) in which these transcribed regions (defined here as *ori*) are removed (Fig. 4A). Both derivatives have normal lytic growth and amplify to high titer (Fig. 4B; data not shown). Using a similar approach to that described above for Miko (Fig. 3C), AlmaΔ*ori* appears unaltered in its lysogenic establishment, and similar numbers of M. smegmatis colonies were recovered on Alma- and AlmaΔ*ori-*seeded plates (Table 2); however, the AlmaΔ*ori*-derived colonies are small and very slow growing compared to Alma-derived colonies (Fig. 3C). When the AlmaΔ*ori*-derived colonies were restreaked, the densest part of the streak failed to grow (as seen for Miko [Fig. 3]), but a mixture of very small and larger isolated colonies were observed (Fig. 4C). Upon further testing, most of the large colonies were nonlysogenic, whereas the small colonies released phage and appeared to be lysogens (Fig. 4D and E; Table 2); nonlysogenic derivatives were recovered only rarely using wild-type Alma. These data suggest that all or part of the deleted region is required for prophage replication. Attempts to recover lysogens from regions where Alma and AlmaΔ*ori* phages were spotted on M. smegmatis lawns support similar conclusions (Fig. S2). Although neither LadyBird nor LadyBird*Δori* lysogens could be recovered from phage-seeded plates, streaking from infected areas of M. smegmatis lawns yielded results similar to the results with Alma, and the LadyBird*Δori* survivors are not stably lysogenic (Fig. S2). We conclude that these small transcribed regions are required for prophage stability.

10.1128/mBio.00385-20.5 FIG S1 Transcriptomic profile of the LadyBird lysogen. RNA was isolated from a LadyBird lysogen in the log phase of growth, and libraries were generated and sequenced using the Illumina platform. The reads per base are plotted along a Phamerator map of LadyBird, with reads aligning to the top strand shown in blue and reads mapping to the bottom strand shown in red. Robust expression of the immunity repressor gene (*79*) and *parAB* (*35* and *36*) is observed, as well as the opposite strand RNA proximal to *parAB* (labeled “*ori*”). Low expression of left-arm structural genes indicates some spontaneous induction. Download FIG S1, PDF file, 0.2 MB. Copyright © 2020 Wetzel et al. 2020 Wetzel et al. This content is distributed under the terms of the Creative Commons Attribution 4.0 International license.

10.1128/mBio.00385-20.6 FIG S2 *ori* is necessary to form stable lysogens of Alma and LadyBird. (A) Representative streaks (*n* = 3) from areas of infection of Alma (right) and AlmaΔ*ori* (left) on an M. smegmatis lawn. (B) Spontaneous phage release from three individual colonies per streak. Individual colonies were patched onto solid media (left) and an M. smegmatis lawn (center). The original infecting phage per streak plate is shown as a grid (right). (C) Susceptibility of colonies to phage superinfection. Liquid cultures were grown from one patch per original area of infection and tested for its susceptibility to Alma, AlmaΔ*ori*, Miko (A2), and a control phage Crossroads (L2). A representative example of each is shown. (D) Representative streaks (*n* = 3) from areas of infection of LadyBird (right) and LadyBirdΔ*ori* (left) on an M. smegmatis lawn. (E) Spontaneous phage release from three individual colonies per streak. Individual colonies were patched onto solid media (left) and an M. smegmatis lawn (center). The original infecting phage per streak plate is shown as a grid (right). (F) Susceptibility of colonies to phage superinfection. Liquid cultures were grown from one patch per original area of infection and tested for its susceptibility to LadyBird, LadyBirdΔ*ori*, Alma (A9), and a control phage Crossroads (L2). A representative example of each is shown. Download FIG S2, PDF file, 0.9 MB. Copyright © 2020 Wetzel et al. 2020 Wetzel et al. This content is distributed under the terms of the Creative Commons Attribution 4.0 International license.

### Phage RepA and non-RepA origins support extrachromosomal autonomous replication.

**FIG 5:**
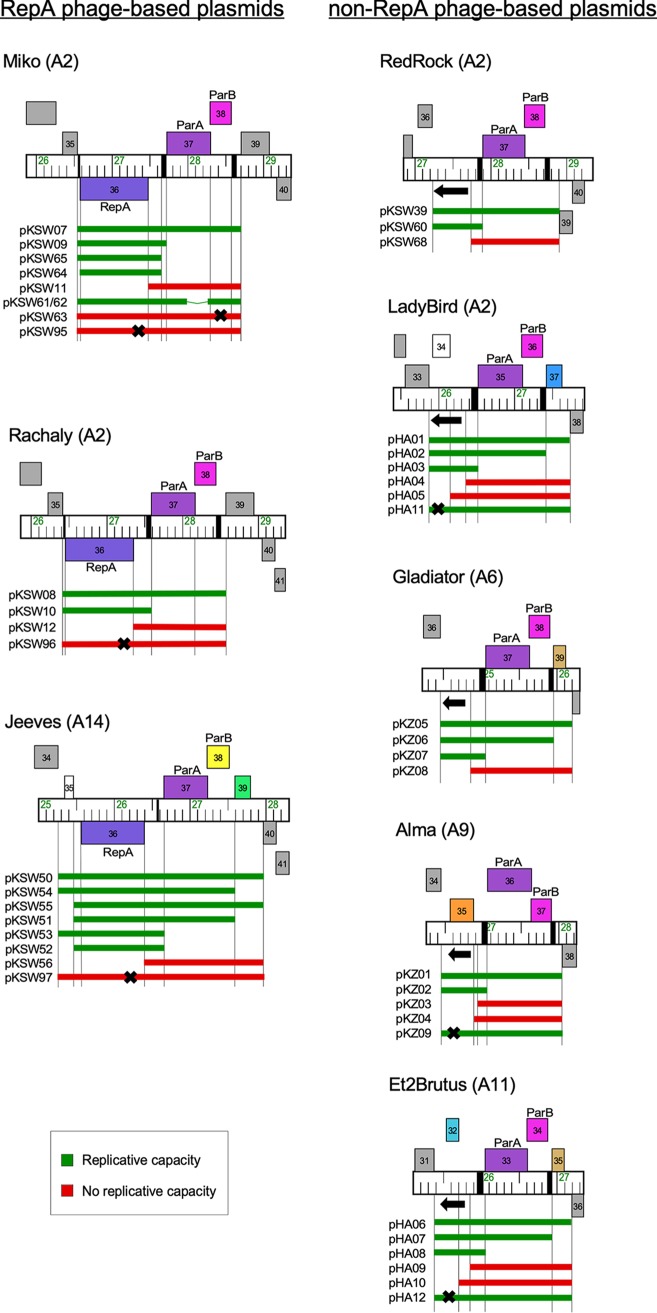
Phage genome segments that support autonomous replication. Segments of eight phage genome maps are shown with relevant genes labeled; the locations of *parS* repeats are indicated by black boxes on the genome ruler. A black arrow indicates the location and transcription direction of the noncoding RNA implicated in replication initiation. Bars underneath each map indicate a genome segment inserted into the nonreplicating vector pMOS-Hyg and then electroporated into M. smegmatis mc^2^155. Replication-proficient plasmids efficiently transforming M. smegmatis (>10^4^ CFU/μg DNA) are shown in green, and those that fail to transform are shown in red. A black X indicates the position of a stop codon introduced by mutagenesis.

To further characterize the phage components required for autonomous replication, we constructed a series of recombinant plasmids carrying segments of RepA phages Miko, Rachaly, and Jeeves and segments of the non-RepA phages, RedRock, LadyBird, Gladiator, Alma, and Et2Brutus, into a vector (pMOS-Hyg) incapable of replicating in M. smegmatis. For the RepA phages, initial recombinant plasmids contained the regions encompassing *repA*, *parA*, *parB*, and included the *parS* sites (Fig. 5). For the non-RepA phages, the initial recombinants included *parA*, *parB*, the *parS* sites, as well as the ∼600-bp region upstream of *parA* carrying the putative *ori*, and one or two of the closely linked ORFs (Fig. 5). All of the plasmids (pKSW07, pKSW08, pKSW50, pKSW39, pHA01, pKZ05, pKZ01, and pHA06, carrying segments from phages Miko, Rachaly, Jeeves, RedRock, LadyBird, Gladiator, Alma, and Et2Brutus, respectively) were able to transform M. smegmatis mc^2^155 with efficiencies similar to those for a control plasmid (pCCK38) containing *oriM* (Table 3). We note that the efficient transformation of M. smegmatis with the RedRock-derived plasmid pKSW39 (Fig. 5) differs from prior reports that a similar phage DNA fragment did not support extrachromosomal replication (16). The primary differences between these constructs is the orientation of the RedRock insert in relation to distinct antibiotic resistance genes (against hygromycin instead of kanamycin), suggesting that the juxtaposition of vector sequences can have a strong impact on replicon functionality.

**TABLE 3:** Transformation efficiencies of phage-based plasmids in M. smegmatis

Plasmid	Phage^*a*^	Feature(s)	Transformation efficiency (CFU/μg of DNA)
pCCK38	N/A	*oriM*	1.2 × 10^5^
pHA06	Et2Brutus	*ori + parABS*	1.1 × 10^5^
pHA08	Et2Brutus	*ori*	3.2 × 10^4^
pKZ05	Gladiator	*ori + parABS*	9.6 × 10^4^
pKZ07	Gladiator	*ori*	1.2 × 10^5^
pHA01	LadyBird	*ori + parABS*	6.5 × 10^4^
pHA03	LadyBird	*ori*	1.2 × 10^5^
pKZ01	Alma	*ori + parABS*	1.0 × 10^5^
pKZ02	Alma	*ori*	1.3 × 10^5^
pKSW39	RedRock	*ori + parABS*	8.9 × 10^4^
pKSW60	RedRock	*ori*	6.6 × 10^4^
pKSW07	Miko	*repA + parABS*	9.0 × 10^4^
pKSW09	Miko	*repA*	1.0 × 10^5^
pKSW08	Rachaly	*repA + parABS*	9.7 × 10^4^
pKSW10	Rachaly	*repA*	8.1 × 10^4^
pKSW50	Jeeves	*repA + parABS*	1.1 × 10^5^
pKSW52	Jeeves	*repA*	8.4 × 10^4^
pMOS-Hyg	N/A	None	0

a N/A, not applicable.

To determine whether the *parABS* partitioning systems are required for autonomous replication, we constructed deletion derivatives of the parental plasmids in which *parA* and *parB* are removed (Fig. 5). All three of the RepA phage-derived plasmids and all five of the non-RepA phage-derived plasmids lacking *parABS* efficiently transformed M. smegmatis, and the *parABS* cassettes are clearly not required for autonomous replication (Fig. 5). These and similar plasmids in which one or more of the flanking ORFs are removed similarly show that these are also not required for replication (Fig. 5).

For the three RepA phages, *repA* and the flanking intergenic regions are sufficient for replication of plasmids pKSW09, pKSW10, and pKSW52, derived from Miko, Rachaly, and Jeeves, respectively (Fig. 5). We further characterized these by constructing additional derivatives and testing their ability to transform M. smegmatis. These experiments showed both that the *parS* sites are not required (e.g., pKSW64 for Miko) and that interruption of the *repA* open reading frame by introduction of an early translation termination codon (in plasmids pKSW95, pKSW96, and pKSW97 for Miko, Rachaly, and Jeeves, respectively) eliminates the replication capacity (Fig. 5); reversion to the wild-type *repA* sequence restored transformation ability (data not shown). The *parABS* systems alone in the absence of *repA* do not support replication, as expected (Fig. 5) (16). For Miko, the minimum segment shown to support replication contains *repA* and 171 bp of upstream sequence (Fig. 5).

For the non-RepA phages, plasmids carrying ∼600 bp to the left of *parA* transform M. smegmatis efficiently (Table 3) and autonomously replicate. In two of these (plasmids pKSW60 and pKZ07 from RedRock and Gladiator, respectively), there are no predicted ORFs, whereas phages LadyBird, Alma, and Et2Brutus have a predicted rightward-transcribed ORF in this region (genes *34*, *35*, and *32*, respectively). Removal of regions containing these ORFs results in loss of transformation (Fig. 5), but the ORFs themselves are not required, because introduction of early translation termination codons does not prevent replication, although the transformants grow somewhat slower than their parental counterparts (data not shown). The reason for reduced growth of these mutant plasmids is unclear, but reversion back to the wild-type sequence restored normal transformation and colony growth. Together, these observations are consistent with the conclusion that the non-RepA phages do not require protein products for replication and that they use RNAs to initiate autonomous replication.

The five non-RepA phage origin of replication regions (defined by the ∼600-bp regions upstream of *parA* sufficient for autonomous replication) vary in relatedness at the nucleotide level (Table 4). Alma and LadyBird are the most similar with ∼71% average nucleotide identity (ANI), and additional pairwise comparisons between RedRock, Alma, Gladiator, and LadyBird range from 62% to 65% ANI (Table 4). The region in Et2Brutus is more distantly related and has between 41% (RedRock) and 49% (Gladiator) ANI. Moreover, there is no open reading frame shared between these phages that is conserved and could potentially be involved in replication.

**TABLE 4:** Percent nucleotide identity of putative replication origins of non-RepA phages

Phage (subcluster)	Coordinates	% nucleotide identity				
		Et2Brutus	Gladiator	RedRock	LadyBird	Alma
Et2Brutus (A11)	25381−26052	100				
Gladiator (A6)	24464−25062	49.0	100			
RedRock (A2)	27232−27897	41.4	62.3	100		
LadyBird (A2)	25875−26522	48.9	62.5	64.4	100	
Alma (A9)	26448−27060	44.0	62.5	65.5	71.0	100

### Roles of *parABS* in plasmid maintenance.

**FIG 6:**
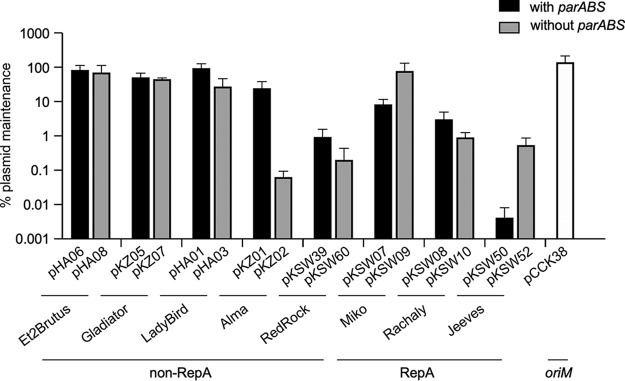
Maintenance of plasmids without selection. M. smegmatis transformants with plasmids (as indicated) were grown in liquid culture with selection to saturation and then serially passaged for a total of ∼40 generations without selection. The percentage of plasmid maintenance was determined by plating serial dilutions of culture on solid media with and without selection. Data represent the mean values from four independent cultures, and error bars represent one standard deviation.

The *parABS* cassette is not necessary for autonomous replication of any of the plasmids tested here, but it is likely required for plasmid maintenance as described for the RedRock *parABS* cassette (16); it also could play a regulatory role in replication. To further explore the roles of *parABS*, we measured the stability of autonomously replicating plasmids and the impact of removal of *parABS* (Fig. 6). Somewhat surprisingly, the stability of plasmids containing *parABS* varied substantially in the absence of selection, varying from being well-maintained (LadyBird) to very unstable (Jeeves). For two of the non-RepA phage derivatives (from Ladybird and Alma), removal of *parABS* resulted in increased plasmid loss as expected, but there was little impact on those derived from Et2Brutus or Gladiator (Fig. 6).

Surprisingly, plasmids pKSW07 and pKSW50 (derived from RepA phages Miko and Jeeves, respectively) are not only very poorly maintained, but removal of *parABS* results in substantial increases in plasmid retention (Fig. 6). We reasoned that a plausible explanation for this paradox is that plasmid copy numbers may have changed from regulatory consequences of removing *parABS*. To determine this, the plasmid copy numbers were measured by whole-genome sequencing of bacterial cultures grown with antibiotic selection (Table 5). We found that the phage-based plasmids with the *parABS* cassettes had copy numbers ranging from 0.4 to 2.1, with the RepA phage-based plasmids having 0.4 to 0.8 copies/cell, and the non-RepA phage-based plasmids having one or two copies/cell (Table 5). Cultures carrying plasmid pKSW50 grow notably slower than other cultures (data not shown), which is likely related to its low average copy number (0.4 copies/cell), reflecting a substantial proportion of nonviable cells when growing in the presence of antibiotic. When *parABS* is removed from the plasmids, copy numbers vary widely, from 0.8/cell (RedRock) to 16.4/cell (Et2Brutus) (Table 5).

**TABLE 5:** Copy number of phage-based plasmids

Plasmid	Phage	Feature(s)	Copy no.
pCCK38	N/A	*oriM*	15.6
pHA06	Et2Brutus	*ori + parABS*	2.1
pHA08	Et2Brutus	*ori*	16.4
pKZ05	Gladiator	*ori + parABS*	1.8
pKZ07	Gladiator	*ori*	7.6
pHA01	LadyBird	*ori + parABS*	1.9
pHA03	LadyBird	*ori*	5.4
pKZ01	Alma	*ori + parABS*	1.2
pKZ02	Alma	*ori*	1.8
pKSW39	RedRock	*ori + parABS*	1
pKSW60	RedRock	*ori*	0.8
pKSW07	Miko	*repA + parABS*	0.8
pKSW09	Miko	*repA*	11.7
pKSW08	Rachaly	*repA + parABS*	0.8
pKSW10	Rachaly	*repA*	2
pKSW50	Jeeves	*repA + parABS*	0.4
pKSW52	Jeeves	*repA*	2.4

The basis for the change in copy number is unclear, but could result either from changes in RepA/*ori* expression, or from *parS*-associated handcuffing or other regulatory mechanisms (41). However, the copy number variation likely accounts for the observed patterns of plasmid stability. First, the most well-maintained plasmids lacking *parABS* have the highest copy numbers (Et2Brutus,16.4 copies/cell), and the least well-maintained have much lower copy numbers (RedRock, 0.8 copies/cell; Alma, 1.8 copies/cell; Table 5); with higher copy numbers, production of plasmid-less cells at division is reduced, especially without a partitioning system. Second, plasmids containing *parABS* typically have lower copy numbers than their cognate parental plasmids, with the exception of RedRock, where there is little difference (Table 5). In systems such as in Gladiator, where the *parABS* cassette is not evidently contributing to maintenance, it enables a lower-copy-number plasmid (pKZ05) to be maintained similarly to a higher-copy-number plasmid (pKZ07). Nonetheless, it is surprising that many of these phage-derived plasmids are not well-maintained even with inclusion of the *parABS* cassette (Table 5). Although additional regulation through the phage repressor, which is encoded by an unlinked gene, is a possibility, we note that the replication/partitioning regions are largely devoid of predicted repressor binding sites; only Rachaly and Jeeves have such sites within or flanking *parABS* (42). Because vector context appears to be important, as illustrated by the behaviors of RedRock-derived plasmids (see above), stabilities and copy numbers of the recombinant plasmids may not fully reflect their parent prophages. For RedRock, LadyBird, Alma, and Et2Brutus, the plasmid copy numbers (one or two copies/cell) are similar although modestly lower than the cognate prophage copy numbers (Tables 1 and 5).

The behaviors and stabilities of this series of plasmids raise the question as to whether the *parABS* cassettes, particularly for RepA phages Miko and Rachaly, are active in partitioning at all. To address this, we used a similar strategy to that described previously to characterize the RedRock *parABS* system, which dramatically stabilizes an *oriM* plasmid expressing mCherry (pLO87) that is very unstable in the absence of selection (16). Recombinant versions of plasmid pLO87 carrying the *parABS* cassettes of Miko and Rachaly confer stability similar to that observed for RedRock *parABS* (16), and increased plasmid retention from <1% to >80% retention over ∼40 generations of unselected growth (Table 6). The reason why the pLO87-derived plasmids are more stable than the plasmids carrying the phage-derived replication systems is unclear, but perhaps is influenced by the vector backbone and vector genes. We note that when the Miko and Rachaly phage cassettes (including *repA* and *parABS* cassettes) are inserted into a different nonreplication plasmid vector, pMD04 (43), we observed similar stabilities (16.6% and 8.6% retention over 40 generations) to their cognate plasmids pKSW07 and pKSW08 (Table 5).

**TABLE 6:** Maintenance of plasmids containing *mCherry*, *oriM*, and phage *parABS*

Plasmid	Phage	Feature(s)	% maintenance^*a*^
pLO87	N/A	N/A	0
pMO01	RedRock	*parABS*	91.0
pKSW35	Miko	*parABS*	96.1
pKSW36	Rachaly	*parABS*	90.0
pKSW37	Miko	*parABS + gp39*	89.2
pKSW38	Rachaly	*parABS + gp39*	81.4

a Percentage of colonies carrying plasmid as determined by fluorescence after 40 generations of unselected growth.

Further evidence for the functionality of *parA* and *parB* in the context of the Miko-derived plasmids is provided by additional plasmid derivatives in which the genes have been interrupted or inactivated (Fig. 5). An early translational termination mutation in Miko *parB* (plasmid pKSW63) results in a nontransformation phenotype, perhaps due to *parA* overexpression or mislocalization, a phenotype similar to that reported previously for RedRock (16). Two plasmid derivatives containing separate in-frame deletions of Miko *parA* (pKSW60 and pKSW61) are competent to transform M. smegmatis but form extremely small colonies, consistent with very poor plasmid retention (data not shown). These observations suggest that the Miko *parABS* system is functional in plasmid pKSW07, even though it does not fully promote plasmid maintenance.

### Compatibility of prophage origin plasmids.

**FIG 7:**
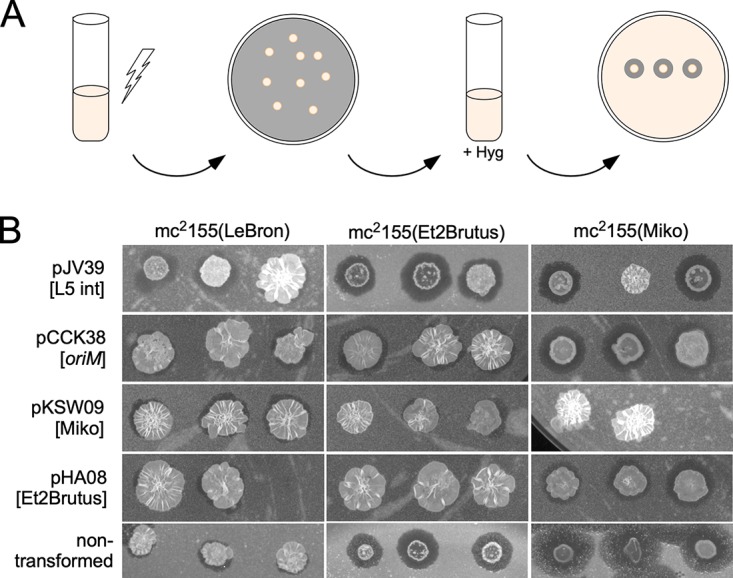
Compatibility of phage-based plasmids and prophages. (A) Scheme to test prophage origin compatibility. M. smegmatis mc^2^155 lysogens of phages LeBron, Et2Brutus, and Miko were transformed with various phage-based plasmids. The resulting colonies were grown in liquid culture with selection for the plasmid and then tested for prophage maintenance by spotting cultures onto lawns of M. smegmatis and observing spontaneous phage release. (B) The compatibilities of LeBron, Et2Brutus, and Miko prophages with plasmids pJV39, pCCK38, pKSW09, and pHA08, as measured by prophage maintenance. These data are a subset of data shown in Table 6. Three transformants were grown per transformation per experiment; missing spots in figure indicate transformant cultures that had not yet grown to saturation at the time of analysis.

Plasmids with common replication systems are typically incompatible as they compete for the replication machinery; therefore, we tested compatibility of a subset of these systems with each other and with the commonly used *oriM* plasmids derived from pAL5000. For this assay, one replicon partner was plasmid pKSW09 (Miko), pKSW52 (Jeeves), pHA08 (Et2Brutus), pKZ07 (Gladiator), or control plasmid pCCK38 (containing *oriM*) or pJV39 (with the L5 *attP*-*int* integration apparatus), and the other was a prophage of Miko, Et2Brutus, or LeBron (an unrelated integrating phage) carried by a lysogenic M. smegmatis strain. The plasmids (which lack *parABS* systems that could independently influence compatibility) were transformed into the lysogens, liquid cultures were grown with plasmid selection, and prophage maintenance was determined by spontaneous phage release (Fig. 7A). If a given prophage and a particular plasmid are compatible, then we expected to see prophage loss that is no greater than in the absence of the plasmid or a vector control (Fig. 7). In contrast, incompatibility would lead to prophage loss and a greater proportion of nonlysogens.

Using a LeBron lysogen as a control, we observed stable prophage maintenance in transformants of all of the tested plasmids (Fig. 7B; Table 7), showing that all of the combinations of the LeBron prophage and plasmid are compatible. Transformants of an Et2Brutus lysogen, carrying the unrelated integrating vector pJV39 also maintain their prophage, but pHA08 transformants (Et2Brutus *ori*) efficiently lose the prophage due to incompatibility (Table 7). The Et2Brutus prophage is fully compatible with plasmids pKSW52 and pKSW09 from Jeeves and Miko, respectively, but is at least partially incompatible with pKZ07 (Gladiator) (Table 7). The Miko prophage is stable in nontransformed cells but is seemingly antagonized by the integrating vector pJV39—perhaps resulting from growth in the presence of hygromycin—leading to substantial prophage loss (Table 7). Transformants with pKSW09 (Miko) are more unstable, as anticipated, but HA08 (Et2Brutus) is relatively well-tolerated and is likely compatible; Miko prophage loss is also observed with pKZ07 (Gladiator) and pKSW02 (Jeeves), but at levels similar to that of the pJV39 control. Both the Miko and Et2Brutus prophages appear to be compatible with the *oriM* plasmid, and prophage loss is not evidently greater than with the control plasmids. These observations suggest that both Miko- and Et2Brutus-derived plasmids can be used compatibly with *oriM* plasmids in mycobacterial genetics and that at least Miko- and Et2Brutus-derived plasmids can be used together without interference.

**TABLE 7:** Compatibility as measured by percent maintenance of prophage with plasmid selection

Prophage	% maintenance of prophage with plasmid selection						
	pCCK38	pJV39	Jeeves (pKSW52)	Miko (pKSW09)	Et2Brutus (pHA08)	Gladiator (pKZ07)	No plasmid
LeBron	83.3	100	100	100	100	100	100
Miko	58.3	41.6	33	22.2	83.3	33	100
Et2Brutus	100	100	100	91.6	0	66.67	100

### Host range of prophage origin plasmids.

We tested plasmid derivatives for each of these systems (with the exception of Rachaly) for their ability to transform M. tuberculosis mc^2^7000. Initially, we used plasmids lacking *parABS* (i.e., pKSW09, pKSW52, pKSW60, pHA03, pKZ07, pKZ02, and pHA08 from Miko, Jeeves, RedRock, LadyBird, Gladiator, Alma, and Et2Brutus, respectively), and all except pKSW09 and pKSW52 (Miko and Jeeves) successfully transformed with frequencies of >10^4^ CFU/μg DNA (Table 8). Colony sizes varied, with pHA03 transformants (LadyBird) yielding the largest colonies and pHA08 (Et2Brutus) yielding the smallest (data not shown). Because pKSW09 and pKSW52 gave no transformants at all, we tested the parent *parABS*-containing plasmids pKSW07 and pKSW50, each of which efficiently transforms M. tuberculosis mc^2^7000 (Table 8). Thus, for these two systems, the *parABS* partitioning system is required for M. tuberculosis transformation, a notable departure from their behaviors in M. smegmatis. We also tested the ability of the phage-based plasmids to transform G. terrae 3612. We observed robust transformation frequencies (>10^5^) for plasmids pKSW09 (Miko), pKZ07 (Gladiator), and pHA08 (Et2Brutus), whereas pHA03 (LadyBird), pKZ02 (Alma), pKSW60 (RedRock), and pKSW52 (Jeeves) did not transform (Table 8); Rachaly-based plasmids were not tested. In contrast to our findings for M. tuberculosis, inclusion of the partitioning cassette did not confer the ability to transform *G. terrae* to the nontransforming phage-based plasmids (Table 8). We note that subcluster A15 contains 13 phages, all of which infect G. terrae, and all of which code for a *parABS* system and are in the non-RepA phage category.

**TABLE 8:** Transformation efficiencies of phage-based plasmids in other *Actinobacteria*

Phage	Feature(s)	Plasmid	Transformation efficiency (CFU/μg of DNA)	
			M. tuberculosis mc^2^7000	*G. terrae* 3612
Et2Brutus	*ori*	pHA08	>10^4^	>10^5^
Gladiator	*ori*	pKZ07	>10^4^	>10^5^
LadyBird	*ori + parABS*	pHA01	Not tested	0
	*ori*	pHA03	>10^4^	0
Alma	*ori + parABS*	pKZ01	Not tested	0
	*ori*	pKZ02	>10^4^	0
RedRock	*ori + parABS*	pKSW39	Not tested	0
	*ori*	pKSW60	>10^4^	0
Miko	*repA + parABS*	pKSW07	>10^4^	Not tested
	*repA*	pKSW09	0	>10^5^
Jeeves	*repA + parABS*	pKSW50	>10^4^	0
	*repA*	pKSW52	0	0
		pMOS-Hyg	0	0

## DISCUSSION

We have described here the putative replication origins and partitioning functions of a series of temperate mycobacteriophages whose prophages are maintained extrachromosomally. Although more than 100 such phages have been reported, only a minority (5%) use a RepA-like initiator protein like that of the prototype P1 prophage. We have demonstrated that RepA is both required and sufficient for autonomous replication, and the *cis*-acting *ori* sequences presumably lie within or immediately adjacent to *repA*. However, most of the autonomously replicating phages do not have *repA*, there are no identifiable protein-coding genes, and it is likely that they use the transcribed RNA to initiate replication. A region expressing these RNAs is necessary and sufficient for autonomous replication.

Mapping and characterizing these prophage replication origins are confounded by differences in the behaviors of related systems derived from different phages, necessitating inclusion of many different systems in the analysis. This is especially notable in the variations in maintenance and copy numbers of recombinant plasmids, and it is likely that non-phage, vector-derived genes or transcripts influence these properties. Nonetheless, by analyzing a repertoire of RepA phages and non-RepA phages, we show that the prophages and prophage-derived plasmids replicate at low copy numbers and that *parABS* promotes plasmid maintenance. Furthermore, at least a subset of these systems provide a new suite of plasmid vectors for use in actinobacterial genetics that offer stably maintained low-copy-number plasmids capable of replicating in both fast- and slow-growing mycobacteria as well as other actinobacterial strains such as *Gordonia*. These are compatible with pAL5000 *oriM* plasmids, facilitating the construction of complex recombinant strains. Although different phages and plasmids may be optimal for specific applications, we note that the Et2Brutus plasmids have the desirable properties of good retention without selection, compatibility with a variety of origins, and broad host range.

The observation that phage-derived DNA segments containing the replication origin and the partitioning functions behave differently in different contexts is quite striking. There are several notable and informative observations. First, prior attempts to characterize the replication system of the non-RepA phage RedRock were discouraging, as insertion of an *ori*-*parABS* cassette into a vector did not promote replication and transformation of M. smegmatis (16). However, inserting the same RedRock DNA cassette into a similar vector backbone (to give pKSW39) but in a different orientation to a distinct antibiotic resistance gene (against hygromycin instead of kanamycin) results in efficient transformation and replication at a copy number similar to that of the prophage (Table 5) (16). Nonetheless, this plasmid is not stably maintained in the absence of selection, even though the *parABS* system should be fully functional (Table 6) (16), and removing it further reduces stability (Fig. 6). Second, it is notable that the Miko *repA*-*parABS* cassette facilitates efficient transformation of M. smegmatis and replicates at low copy number (Table 5), although it is quite unstable in the absence of selection (Fig. 6). Removal of *parABS* results in a striking increase in stability, seemingly facilitated by a 10-fold increase in plasmid copy number. In general, these behaviors suggest that replication is tightly regulated with a potential interaction between the partitioning and replication systems.

A further oddity is the unexpected dependence on the *parABS* cassette of Miko and Jeeves for transformation of M. tuberculosis. Both use a RepA-dependent replication system, and neither requires *parABS* for M. smegmatis transformation. In M. smegmatis, RepA plasmids lacking *parABS* have higher copy numbers than RepA plasmids that contain *parABS* do (Table 5) and are more stable. It is unclear why such changes would result in the inability to replicate in M. tuberculosis. However, we note that at least in some contexts, RepA expression is likely toxic, as plasmids expressing Miko RepA from the strong constitutive *hsp60* promoter do not transform M. smegmatis (data not shown). Nonetheless, a variety of plasmids with different origins are available for use as M. tuberculosis plasmid vectors, and their compatibility with *oriM* plasmids, integrating, and *parABS* phage-based plasmids represents a substantial expansion of opportunities for M. tuberculosis genetics. The Gladiator-, Alma-, and Miko-derived plasmids also replicate in *Gordonia*, and it is likely all or many of the plasmids described here will be useful for genetic analysis of other actinobacterial strains, including nontuberculosis mycobacteria (NTM) pathogens such as M. abscessus and Mycobacterium avium. Additionally, the instability of some of these plasmids could be utilized to develop transient transposon delivery systems for various actinobacterial strains.

The extrachromosomally replicating temperate actinobacteriophages are almost exclusively found within cluster A; the exception, *Streptomyces* phage pZL12, is a singleton phage with no close relatives (44). Cluster A is exceptionally large (>600 individual phages), so we cannot exclude the possibility that other extrachromosomally replicating temperate phages will not be found in other less-well-sampled clusters of temperate actinobacteriophages, all of which are less than a quarter of the size of cluster A. Interestingly, although 50% of the cluster A subclusters have *parABS* phages (Fig. 1), most have only *parABS* phages, whereas subclusters A2, A9, and A12 have both integrating and *parABS* phage members. Because both the integration cassettes of subcluster A2 phages such as those in L5 and D29 (45, 46) as well as the replication partitioning system of subcluster A2 RepA phages are fully functional outside their phage contexts, it seems likely that they can be readily exchanged between the two, and comparison of cluster A2 genomes suggests this has likely occurred in their relatively recent evolutionary pasts. The genomes of phages Lokk and BobSwaget exemplify this, as they have 97% ANI and identical gene content, except for the additional RepA homologue found in Lokk (47). We are not aware of other sets of closely related genomes where this is observed, and note that this would likely not occur with the prototype lambda phage in which the integration apparatus is well-integrated into the overall regulatory circuitry, including dependence on the unlinked *cII* gene for integrase expression (48).

## MATERIALS AND METHODS

### Bacteria and plasmids.

M. smegmatis mc^2^155, M. tuberculosis mc^2^7000, and Gordonia terrae 3612 were grown as described previously (49, 50). To construct phage-based plasmids, genome segments were PCR amplified from phage lysates using Q5 HiFi 2× MasterMix (NEB), and amplicons were inserted into the HindIII-digested vector pMOS-Hyg or XmnI-digested pMD04 (43) using the NEBuilder HiFi assembly kit (NEB); plasmids containing point mutations or deletions were constructed using the Q5 site-directed mutagenesis (SDM) kit (NEB). Transformations used electroporation protocols described previously for *Mycobacterium* (51) and *Gordonia* (49), and transformants were recovered on solid media with antibiotics (pMOS-Hyg, 50 μg/ml hygromycin; pMD04, 20 μg/ml kanamycin) and incubated at 37°C (M. smegmatis and M. tuberculosis) or 30°C (G. terrae). Plasmids used in this study have been compiled in Table S3 and S4 in the supplemental material.

### Construction of mutant bacteriophages.

Phage genomic DNA was extracted from high-titer lysates of LadyBird, Alma, and Miko using phenol-chloroform extraction. Phage DNA was coelectroporated into recombineering M. smegmatis mc^2^155::pJV53 cells (51) with substrate DNA complementary to 250 bp flanking the deletion as previously described (40); genome coordinates and primer sequences are shown in Table S2. Cells were recovered for 3 to 4 h at 37°C and then combined with an M. smegmatis strain containing a plasmid with an anhydrotetracycline (ATc)-inducible CRISPR system targeting unmutated phage and plated on 7H11 with albumin-dextrose-catalase (ADC), kanamycin (Kan), CaCl_2_, and 300 ng/ml ATc. Plaques were screened for deletion via PCR and sequenced as described previously (52).

10.1128/mBio.00385-20.2 TABLE S2 DNA substrates and primers used in phage engineering. Download Table S2, DOCX file, 0.01 MB. Copyright © 2020 Wetzel et al. 2020 Wetzel et al. This content is distributed under the terms of the Creative Commons Attribution 4.0 International license.

10.1128/mBio.00385-20.3 TABLE S3 Plasmids used in this study. Download Table S3, DOCX file, 0.02 MB. Copyright © 2020 Wetzel et al. 2020 Wetzel et al. This content is distributed under the terms of the Creative Commons Attribution 4.0 International license.

10.1128/mBio.00385-20.4 TABLE S4 Mutant plasmids used in this study. Download Table S4, DOCX file, 0.01 MB. Copyright © 2020 Wetzel et al. 2020 Wetzel et al. This content is distributed under the terms of the Creative Commons Attribution 4.0 International license.

The genome of the Miko parent phage used here varies slightly from the published sequence (GenBank accession number MN369748), in that a 153-bp repeat (coordinates 24718 to 24870) in gene *34* (minor tail protein) occurs once, whereas in the published sequence, it occurs twice. On sequencing of LadyBirdΔ*ori*, several base changes were observed in gene *31*, coding for a predicted minor tail protein. These mutations did not alter the instability of the prophage (Fig. S3). AlmaΔ*ori* also had a single base change in gene *17*, coding for a predicted major capsid protein.

10.1128/mBio.00385-20.7 FIG S3 Several point mutations in a minor tail protein gene gp*31* do not alter prophage stability phenotypes. (A) Representative streaks from areas of infection of LadyBird G24044A (right) and LadyBirdΔori G24044A, C24885A (center) and LadyBirdΔori G24044A, G24980A (left) on an M. smegmatis lawn. (B) Spontaneous phage release from three individual colonies per streak, three streaks total. Individual colonies were patched onto an M. smegmatis lawn (top) and solid media (bottom). At least two patches/colony from LadyBird G24044A released phage, indicative of lysogens. Only one patch from LadyBirdΔ*ori* G24044A, C24885A released phage and 0 patches from LadyBirdΔ*ori* G24044A, G24980A released phage, indicative of unstable prophages or nonlysogens. Patches from a streak from a spot of phage buffer on an M. smegmatis lawn are shown for comparison. Download FIG S3, PDF file, 0.8 MB. Copyright © 2020 Wetzel et al. 2020 Wetzel et al. This content is distributed under the terms of the Creative Commons Attribution 4.0 International license.

### Phenotyping mutant bacteriophages.

Serial dilutions of M. smegmatis mc^2^155 in log phase (optical density at 600 nm [OD_600_] of 0.8) were spread on plates seeded with 10^9^ phage particles, or phage buffer as a control, and incubated for 4 days. Well-isolated colonies were streaked to remove phage, and three colonies per streak were patched onto lawns of M. smegmatis mc^2^155 to test for spontaneous phage release. Liquid cultures were grown from patches on solid media to test for susceptibility to the original infecting phage.

### RNA-Seq.

Strand-specific transcription profiles of the LadyBird lysogen were measured by transcriptome sequencing (RNA-Seq) as previously described (53) and viewed using Integrated Genomics Viewer (IGV) (54) (Fig. S2).

### Amino acid and nucleotide alignment and phylogeny.

Amino acid sequences of plasmid and phage RepA proteins (Fig. 2C) were aligned using Clustal Omega (55), and a phylogenetic tree using maximum likelihood (PhyML) was constructed with SeaView (56) and visualized using EvolView (57). Nucleotide sequences for the non-RepA phage origins (Table 3) were aligned using Clustal Omega.

### Plasmid maintenance assays.

M. smegmatis transformants carrying phage-based plasmids were grown with selection to saturation, diluted 1:10,000, and regrown to saturation in antibiotic-free media; this was performed three times for a total of ∼40 generations without selection. The final culture was serially diluted, spotted (10 μl) onto solid media with and without selection, and incubated for 3 days at 37°C. The resulting colonies were counted, and maintenance was calculated as the number of colonies on the plate with selection divided by the number of colonies on the plate lacking antibiotics. This experiment was performed twice with duplicates. To determine maintenance of mCherry-expressing plasmids, the experiment was performed similarly, but retention was measured as the proportion of pink colonies on unselected plates.

### Compatibility of prophage origins.

An M. smegmatis mc^2^155 lysogen of Et2Brutus was described previously (42) and lysogens of LeBron and Miko were made following the same protocol. Electrocompetent cells of the LeBron, Miko, and Et2Brutus lysogens were transformed with plasmids pJV39, pCCK38, pKSW52, pKSW09, pHA08, and pKZ07. The transformed lysogens were grown on hygromycin (Hyg) plates for 3 to 4 days at 37°C. Three colonies were picked from each of these plates, and liquid cultures were grown to saturation with hygromycin at 37°C to select for the plasmid. The liquid cultures were spotted onto lawns of M. smegmatis mc^2^155 and incubated at 37°C. Prophage maintenance was determined by observation of spontaneous phage release from spots of liquid culture. Compatibility was calculated as the percentage of transformed lysogen cultures that maintained the prophage after selection for the plasmid for at least six independent cultures.

### Determination of plasmid copy number.

M. smegmatis transformants carrying phage-based plasmids were grown with selection to log phase (OD_600_ of ∼0.8) and DNA was extracted using phenol-chloroform. The DNA was sequenced using the Illumina platform as previously described (52), and copy number was calculated as the ratio of the average coverage of the plasmid sequence to the M. smegmatis chromosome. Prophage copy number was calculated similarly. For several plasmids, two transformants were evaluated (pCCK38, pKSW50, pKSW52, pKZ05, and pKZ07) showing good repeatability (<5% variation except for the low-copy-number vector pKSW50, which varied by 25%, or 0.1, between replicates); therefore, the remaining transformants were evaluated once.

### Data availability.

All phage genome sequences are available at phagesdb.org. RNA-Seq data for the LadyBird lysogen have been deposited in the Gene Expression Omnibus (GEO) with accession number GSE145724.
