# Supplementary material for: Protein-Mediated and RNA-Based Origins of Replication of Extrachromosomal Mycobacterial Prophages
Source: mBio. 2020 Mar 24;11(2):e00385-20. doi: 10.1128/mBio.00385-20 (PMC7157519; doi:10.1128/mBio.00385-20)
Supplement: TABLE S1 [file mBio.00385-20-st001.docx]

Table S1. Cluster A Actinobacteriophages that contain *parABS*

| **Phage** | **Cluster** | **Subcluster** | **RepA Pham (if present)^1^** | **ParA Pham** | **ParB Pham** |
| --- | --- | --- | --- | --- | --- |
| 20ES | A | A2 | - | 46014 | 101756 |
| 40AC | A | A17 | - | 46014 | 37021 |
| Aliter | A | A9 | - | 46014 | 101756 |
| Alma | A | A9 | - | 46014 | 101756 |
| Aneem | A | A11 | - | 46014 | 101756 |
| AnnaL29 | A | A2 | - | 46014 | 93634 |
| Anon | A | A15 | - | 46014 | 37021 |
| ArcherNM | A | A2 | - | 46014 | 101756 |
| Arissanae | A | A9 | - | 46014 | 93634 |
| Artemis2UCLA | A | A6 | - | 46014 | 101756 |
| Bachome | A | A11 | - | 46014 | 101756 |
| Beemo | A | A9 | - | 46014 | 101756 |
| BengiVuitton | A | A2 | - | 46014 | 101756 |
| Benvolio | A | A2 | - | 46014 | 101756 |
| Blinn1 | A | A6 | - | 46014 | 101756 |
| Blue7 | A | A6 | - | 46014 | 101756 |
| BobSwaget | A | A2 | - | 46014 | 93634 |
| BogosyJay | A | A9 | - | 46014 | 101756 |
| Bowtie | A | A11 | - | 46014 | 101756 |
| Bud | A | A11 | - | 46014 | 101756 |
| Catalina | A | A9 | - | 46014 | 101756 |
| Changeling | A | A2 | - | 46014 | 101756 |
| CloudWang3 | A | A6 | - | 46014 | 101756 |
| Conquerage | A | A9 | - | 46014 | 101756 |
| CRB1 | A | A2 | 23651 | 46014 | 93634 |
| DaVinci | A | A6 | - | 46014 | 101756 |
| EagleEye | A | A16 | - | 46014 | 101756 |
| Ebony | A | A11 | - | 46014 | 101756 |
| Echild | A | A2 | - | 46014 | 37021 |
| Eidsmoe | A | A9 | - | 46014 | 101756 |
| Elephantoon | A | A9 | - | 46014 | 101756 |
| EricB | A | A6 | - | 46014 | 101756 |
| Et2Brutus | A | A11 | - | 46014 | 101756 |
| ExplosioNervosa | A | A9 | - | 46014 | 101756 |
| Fibonacci | A | A11 | - | 46014 | 101756 |
| First | A | A2 | - | 46014 | 101756 |
| Flaverint | A | A11 | - | 46014 | 101756 |
| Gladiator | A | A6 | - | 46014 | 101756 |
| GreedyLawyer | A | A6 | - | 46014 | 101756 |
| Gruunaga | A | A6 | - | 46014 | 101756 |
| Hammer | A | A6 | - | 46014 | 101756 |
| Hexamo | A | A6 | - | 46014 | 101756 |
| HortumSL17 | A | A9 | - | 46014 | 101756 |
| Hutc2 | A | A11 | - | 46014 | 101756 |
| Isiphiwo | A | A6 | - | 46014 | 101756 |
| Jabith | A | A11 | - | 46014 | 101756 |
| Jeeves | A | A14 | 23651 | 46014 | 93634 |
| Jeffabunny | A | A6 | - | 46014 | 101756 |
| JewelBug | A | A6 | - | 46014 | 101756 |
| Jordennis | A | A6 | - | 46014 | 101756 |
| Joselito | A | A11 | - | 46014 | 101756 |
| JSwag | A | A15 | - | 46014 | 93634 |
| KatherineG | A | A15 | - | 46014 | 93634 |
| Kazan | A | A6 | - | 46014 | 101756 |
| Keziacharles14 | A | A9 | - | 46014 | 101756 |
| Kipper29 | A | A6 | - | 46014 | 101756 |
| Koko | A | A6 | - | 46014 | 101756 |
| LadyBird | A | A2 | - | 46014 | 101756 |
| LastResort | A | A15 | - | 46014 | 93634 |
| Lokk | A | A2 | 23651 | 46014 | 93634 |
| LoneWolf | A | A9 | - | 46014 | 101756 |
| Loser | A | A2 | - | 46014 | 101756 |
| Luchador | A | A14 | - | 46014 | 93634 |
| Lucyedi | A | A16 | - | 46014 | 101756 |
| Mabel | A | A11 | - | 46014 | 101756 |
| Maminiaina | A | A9 | - | 46014 | 101756 |
| McFly | A | A6 | - | 46014 | 101756 |
| Miko | A | A2 | 23651 | 46014 | 101756 |
| MinecraftSteve | A | A15 | - | 46014 | 93634 |
| Mulciber | A | A11 | - | 46014 | 101756 |
| Munch | A | A11 | - | 46014 | 101756 |
| Myxus | A | A9 | - | 46014 | 101756 |
| Nedarya | A | A15 | - | 46014 | 101756 |
| Orange | A | A11 | - | 46014 | 101756 |
| PackMan | A | A9 | - | 46014 | 101756 |
| Phaeder | A | A9 | - | 46014 | 101756 |
| Phlei | A | A13 | - | 46014 | 101756 |
| Phonnegut | A | A9 | - | 46014 | 101756 |
| Pioneer | A | A9 | - | 46014 | 101756 |
| Pmask | A | A6 | - | 46014 | 101756 |
| Priamo | A | A6 | - | 46014 | 101756 |
| Priya | A | A9 | - | 46014 | 101756 |
| Qobbit | A | A9 | - | 46014 | 101756 |
| Rachaly | A | A2 | 23651 | 46014 | 101756 |
| Rahalelujah | A | A9 | - | 46014 | 101756 |
| RedRock | A | A2 | - | 46014 | 101756 |
| Refuge | A | A12 | - | 46014 | 53339 |
| ReMo | A | A15 | - | 46014 | 93634 |
| Remus | A | A15 | - | 46014 | 93634 |
| Rosalind | A | A15 | - | 46014 | 93634 |
| Salz | A | A11 | - | 46014 | 101756 |
| Scherzo | A | A9 | - | 46014 | 101756 |
| ShayRa | A | A15 | - | 46014 | 93634 |
| Snape | A | A11 | - | 46014 | 101756 |
| Soups | A | A15 | - | 46014 | 93634 |
| Steamy | A | A12 | - | 46014 | 101756 |
| Strosahl | A | A15 | - | 46014 | 93634 |
| SuperAwesome | A | A6 | - | 46014 | 101756 |
| Toaka | A | A9 | - | 46014 | 101756 |
| ToneTone | A | A6 | - | 46014 | 101756 |
| Tubs | A | A9 | - | 46014 | 101756 |
| Ugenie5 | A | A9 | - | 46014 | 101756 |
| Vanisoa | A | A9 | - | 46014 | 101756 |
| VohminGhazi | A | A6 | - | 46014 | 101756 |
| Waits | A | A15 | - | 46014 | 93634 |
| Wiks | A | A6 | - | 46014 | 101756 |
| WunderPhul | A | A6 | - | 46014 | 101756 |
| Yokurt | A | A6 | - | 46014 | 101756 |
| Zaka | A | A6 | - | 46014 | 101756 |
| Zulu | A | A6 | - | 46014 | 101756 |

^1^Assigned pham numbers according to Actino_Draft Database version 337 accessed January 30, 2020.
