# Supplementary figures and images for: Protein-Mediated and RNA-Based Origins of Replication of Extrachromosomal Mycobacterial Prophages
Source: mBio. 2020 Mar 24;11(2):e00385-20. doi: 10.1128/mBio.00385-20 (PMC7157519; doi:10.1128/mBio.00385-20)

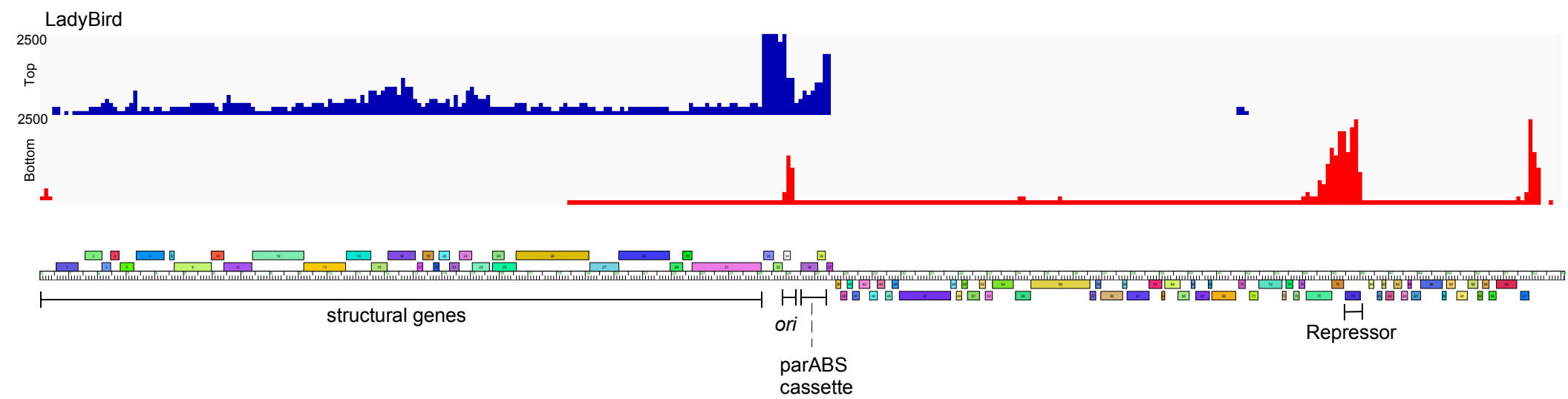

Figure S1

Supplement: FIG S1 [file mBio.00385-20-sf001.pdf]

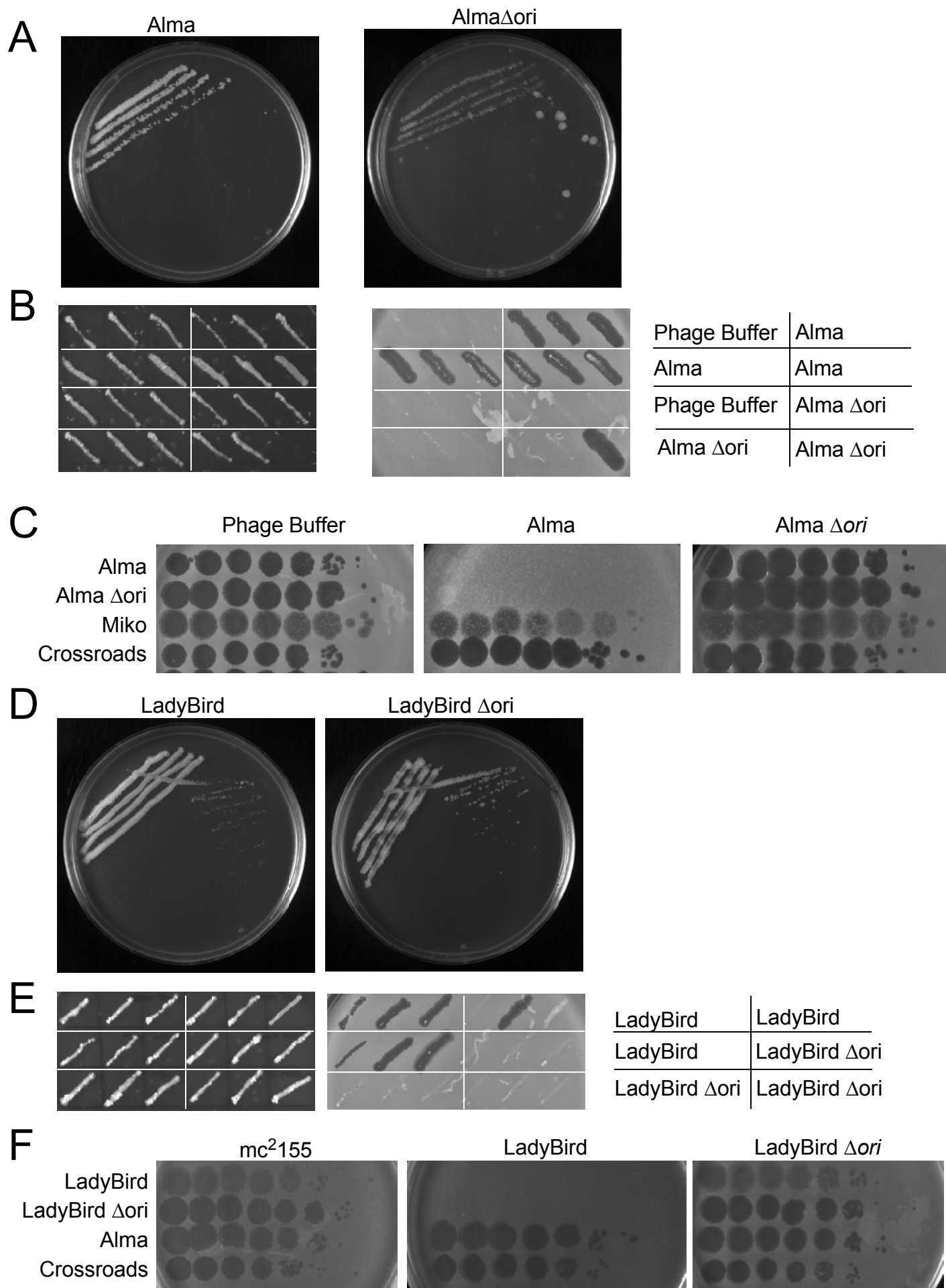

Figure S2

Supplement: FIG S2 [file mBio.00385-20-sf002.pdf]
