# Supplementary material for: Protein-Mediated and RNA-Based Origins of Replication of Extrachromosomal Mycobacterial Prophages
Source: mBio. 2020 Mar 24;11(2):e00385-20. doi: 10.1128/mBio.00385-20 (PMC7157519; doi:10.1128/mBio.00385-20)
Supplement: TABLE S2 [file mBio.00385-20-st002.docx]

Table S2. DNA substrates and primers used in phage engineering

| Phage | gBlock Coordinates | Primers to amplify gBlock | Deletion Coordinates |
| --- | --- | --- | --- |
| Miko ∆*repA* | 26,347-26,596; 27,467-27,716 | MikoKORepAgBFwd: GATCATGAAGTCGATCGCAGCGATG;  MikoKORepAgBRev: GTACTCGAATCCTCGAGGACTAGAGG | 26,597-27,466 |
| Alma ∆*ori* | 26,226- 26,462; 26,960- 27,221 | Alma _V2_gBlock_Fwd: GCGTTTCCGGGCGTACCGT;  Alma ori gBlock new Rev: TCTTCGGCCCACACGGAC | 26,463-26,959 |
| LadyBird ∆*ori* | 25,650- 25,899; 26,300- 26,549 | LadyBird_ori_gBlock_Fwd: AGAGACGGCAAGCACTTCCT;  LadyBird_ori_gBlock_Rev: CGAGATGATCGTTGTCATGCTGGT | 25,900-26,299 |
