# Supplementary material for: Protein-Mediated and RNA-Based Origins of Replication of Extrachromosomal Mycobacterial Prophages
Source: mBio. 2020 Mar 24;11(2):e00385-20. doi: 10.1128/mBio.00385-20 (PMC7157519; doi:10.1128/mBio.00385-20)
Supplement: TABLE S3 [file mBio.00385-20-st003.docx]

Table S3. Plasmids used in this study

| **Plasmid** | **Source Phage** | **Included phage coordinates** | **Included ORFs** | **Vector** | **Resistance Marker** |
| --- | --- | --- | --- | --- | --- |
| pHA01 | LadyBird | 25875 - 27739 | gp34-gp37 | pMOS-Hyg | HygR |
| pHA02 | LadyBird | 25875 - 27423 | gp34-gp36 | pMOS-Hyg | HygR |
| pHA03 | LadyBird | 25875 - 26522 | gp34 | pMOS-Hyg | HygR |
| pHA04 | LadyBird | 26367 - 27739 | gp35-gp37 | pMOS-Hyg | HygR |
| pHA05 | LadyBird | 26153 - 27739 | gp35-gp37 | pMOS-Hyg | HygR |
| pHA06 | Et2Brutus | 25381-27198 | gp32-gp35 | pMOS-Hyg | HygR |
| pHA07 | Et2Brutus | 25381-26936 | gp32-gp34 | pMOS-Hyg | HygR |
| pHA08 | Et2Brutus | 25381-26052 | gp32 | pMOS-Hyg | HygR |
| pHA09 | Et2Brutus | 25853-27198 | gp33-gp35 | pMOS-Hyg | HygR |
| pHA10 | Et2Brutus | 25707-27198 | gp33-gp35 | pMOS-Hyg | HygR |
| pKSW07 | Miko | 26546-28707 | gp36-gp38 | pMOS-Hyg | HygR |
| pKSW08 | Rachaly | 26421-28573 | gp36-gp38 | pMOS-Hyg | HygR |
| pKSW09 | Miko | 26546-27725 | gp36 | pMOS-Hyg | HygR |
| pKSW10 | Rachaly | 26421-27591 | gp36 | pMOS-Hyg | HygR |
| pKSW11 | Miko | 27482-28707 | gp37-gp38 | pMOS-Hyg | HygR |
| pKSW12 | Rachaly | 27357-28573 | gp36-gp37 | pMOS-Hyg | HygR |
| pKSW27 | Miko | 26546-28707 | gp36-gp38 | pMD04 | KanR |
| pKSW28 | Rachaly | 26421-28573 | gp36-gp38 | pMD04 | KanR |
| pKSW35 | Miko | 26546-28707 | gp36-gp38 | pLO87 | KanR |
| pKSW36 | Rachaly | 26421-28573 | gp36-gp38 | pLO87 | KanR |
| pKSW37 | Miko | 26546-29176 | gp36-gp39 | pLO87 | KanR |
| pKSW38 | Rachaly | 26421-29052 | gp36-gp39 | pLO87 | KanR |
| pKSW39 | RedRock | 27232-28911 | gp37-gp38 | pMOS-Hyg | HygR |
| pKSW50 | Jeeves | 25262-27970 | gp35-gp39 | pMOS-Hyg | HygR |
| pKSW51 | Jeeves | 25465-27595 | gp36-gp38 | pMOS-Hyg | HygR |
| pKSW52 | Jeeves | 25465-26657 | gp36 | pMOS-Hyg | HygR |
| pKSW53 | Jeeves | 25262-26657 | gp35-gp36 | pMOS-Hyg | HygR |
| pKSW54 | Jeeves | 25262-27595 | gp35-gp38 | pMOS-Hyg | HygR |
| pKSW55 | Jeeves | 25465-27970 | gp36-gp39 | pMOS-Hyg | HygR |
| pKSW56 | Jeeves | 26400-27970 | gp37-gp39 | pMOS-Hyg | HygR |
| pKSW60 | RedRock | 27232-27897 | none | pMOS-Hyg | HygR |
| pKSW64 | Miko | 26582-27652 | gp36 | pMOS-Hyg | HygR |
| pKSW65 | Miko | 26546-27652 | gp36 | pMOS-Hyg | HygR |
| pKSW68 | RedRock | 27724-28911 | gp37-gp38 | pMOS-Hyg | HygR |
| pKZ01 | Alma | 26448 - 28047 | gp35-gp37 | pMOS-Hyg | HygR |
| pKZ02 | Alma | 26448 - 27060 | gp35 | pMOS-Hyg | HygR |
| pKZ03 | Alma | 26913 - 28047 | gp36-gp37 | pMOS-Hyg | HygR |
| pKZ04 | Alma | 26877 - 28047 | gp36-gp37 | pMOS-Hyg | HygR |
| pKZ05 | Gladiator | 24464 - 26207 | gp37-gp39 | pMOS-Hyg | HygR |
| pKZ06 | Gladiator | 24464- 25956 | gp37-gp38 | pMOS-Hyg | HygR |
| pKZ07 | Gladiator | 24464 - 25062 | none | pMOS-Hyg | HygR |
| pKZ08 | Gladiator | 24863 - 26207 | gp37-gp39 | pMOS-Hyg | HygR |
