# Supplementary material for: Protein-Mediated and RNA-Based Origins of Replication of Extrachromosomal Mycobacterial Prophages
Source: mBio. 2020 Mar 24;11(2):e00385-20. doi: 10.1128/mBio.00385-20 (PMC7157519; doi:10.1128/mBio.00385-20)
Supplement: TABLE S4 [file mBio.00385-20-st004.docx]

Table S4. Mutant plasmids used in this study

| **Plasmid** | **Source Phage** | **Parent plasmid** | **Introduced mutation** |  |
| --- | --- | --- | --- | --- |
| pKSW61 | Miko | pKSW07 | Deletion of insert coordinates 28015-28238 (parA ORF) | |
| pKSW62 | Miko | pKSW07 | Deletion of insert coordinates 27838-28065 (parA ORF) | |
| pKSW63 | Miko | pKSW07 | Mutates #4th codon of parB from GAG to TAG/UAG stop (10th ntide changed); After end of parA reading frame | |
| pHA11 | LadyBird | pHA01 | Mutates 3rd codon of gp34 from GGG to TAG/UAG stop (7th and 8th nucleotides changed) | |
| pKZ09 | Alma | pKZ01 | Mutates 2nd codon of gp35/ ori from GAG to TAG/UAG stop (4th nucleotide changed) | |
| pHA12 | Et2Brutus | pHA06 | Mutates 5th codon of gp32 from GGT to TGA/UGA stop (13th and 15th nucleotides changed) | |
| pKSW95 | Miko | pKSW07 | Mutates 4th codon of repA from TCG to TAG/UAG stop (11th ntide changed) | |
| pKSW97 | Rachaly | pKSW08 | Mutates 4th codon of repA from TCG to TAG/UAG stop (11th ntide changed) | |
| pKSW98 | Jeeves | pKSW50 | Mutates 4th codon of repA from TCG to TAG/UAG stop (11th ntide changed) | |
