# Supplementary material for: Protein-Mediated and RNA-Based Origins of Replication of Extrachromosomal Mycobacterial Prophages
Source: mBio. 2020 Mar 24;11(2):e00385-20. doi: 10.1128/mBio.00385-20 (PMC7157519; doi:10.1128/mBio.00385-20)
Supplement: FIG S3 [file mBio.00385-20-sf003.pdf]

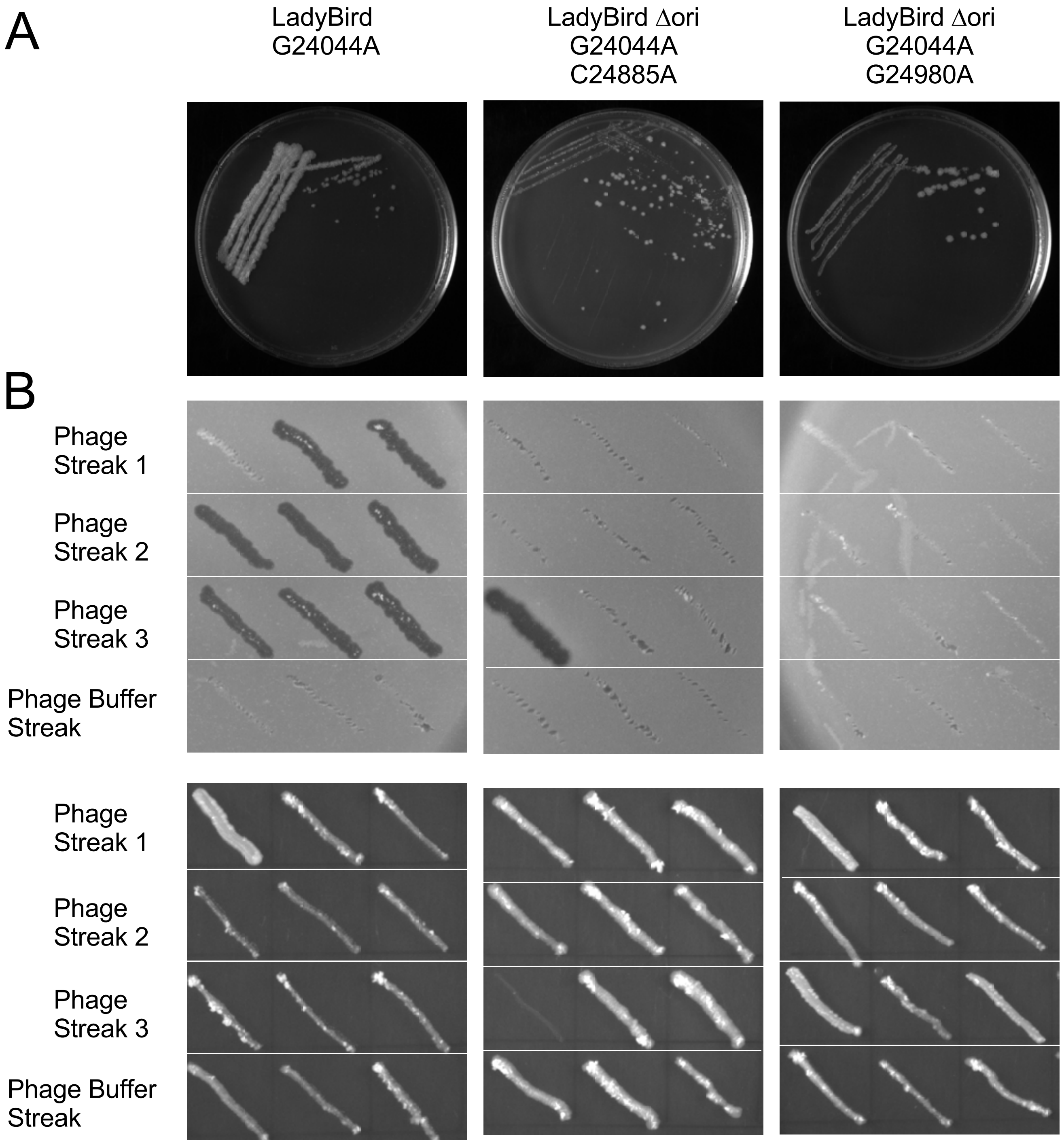
A
LadyBird
G24044A
LadyBird Δori
G24044A
C24885A
LadyBird Δori
G24044A
G24980A
B
Phage
Streak 1
Phage
Streak 2
Phage
Streak 3
Phage Buffer
Streak
Phage
Streak 1
Phage
Streak 2
Phage
Streak 3
Phage Buffer
Streak

Figure S3
